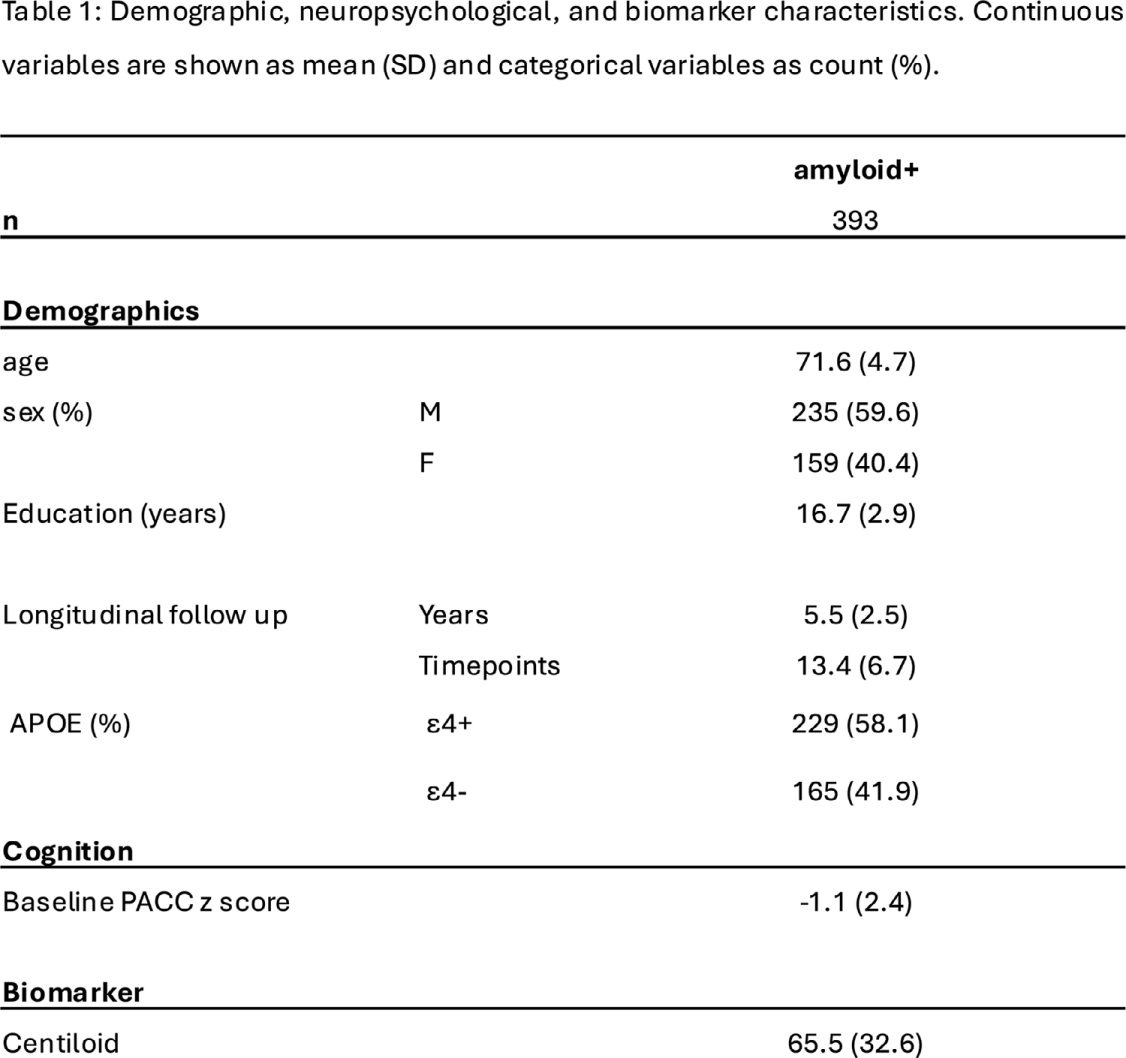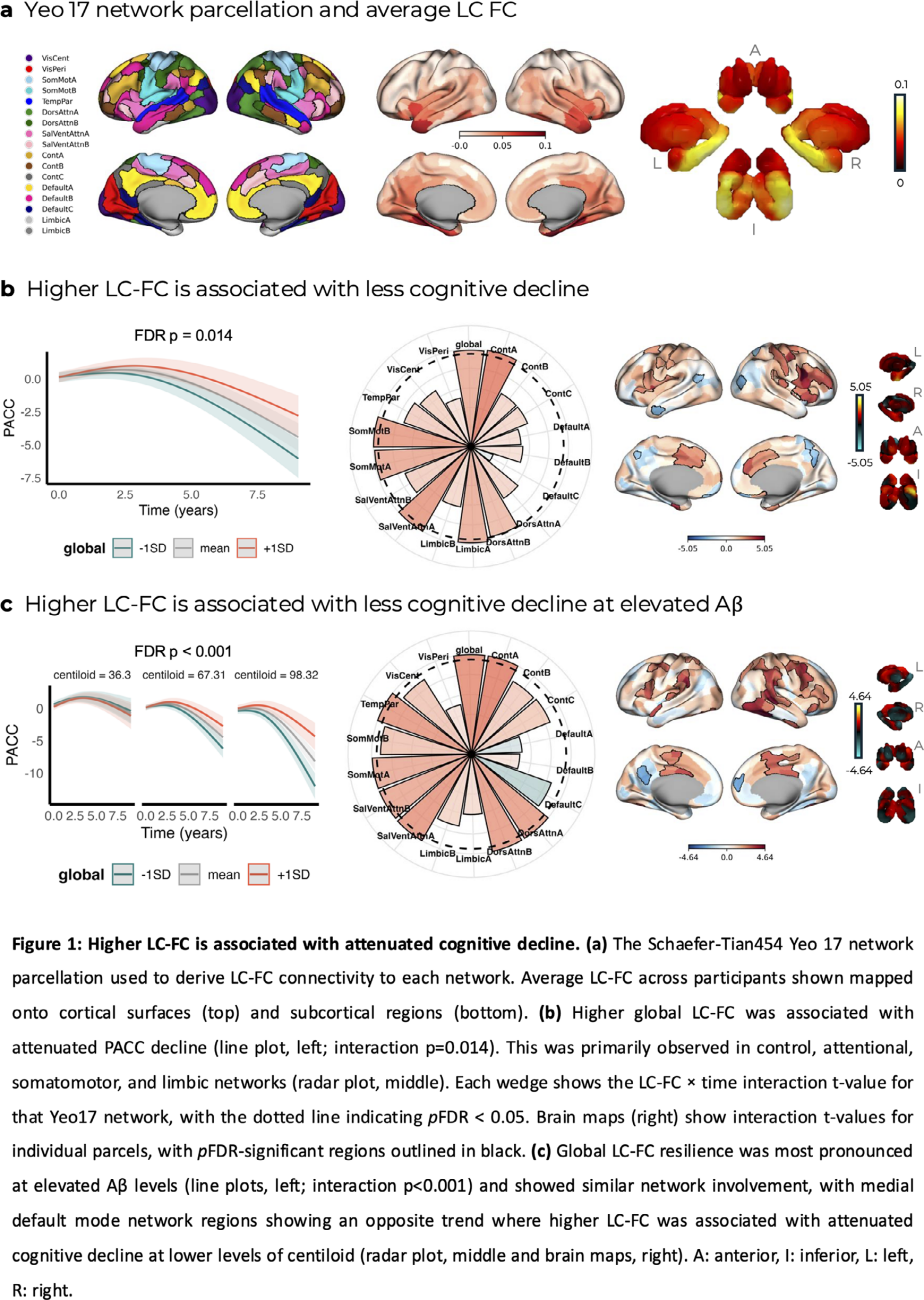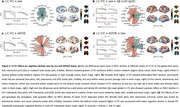# Sex and APOE status moderate locus coeruleus network related resilience in preclinical Alzheimer's disease

**DOI:** 10.1002/alz70856_107257

**Published:** 2026-01-11

**Authors:** Timothy Lawn, Ibai Diez, Elisenda Bueichekú2, Maxime Van Egroo, Gillian T Coughlan, Rachel F. Buckley, Dorene M. Rentz, Keith A. Johnson, Reisa A. Sperling, Jorge Sepulcre, Heidi I.L. Jacobs

**Affiliations:** ^1^ Massachusetts General Hospital and Harvard Medical School, Boston, MA, USA; ^2^ Biobizkaia HRI, Barakaldo, Bizkaia, Spain; ^3^ Massachusetts General Hospital, Harvard Medical School, Boston, MA, USA; ^4^ Yale, New Haven, CT, USA; ^5^ Athinoula A. Martinos Center, Massachusetts General Hospital, Boston, MA, USA; ^6^ Faculty of Health, Medicine and Life Sciences, School for Mental Health and Neuroscience, Alzheimer Centre Limburg, Maastricht University, Maastricht, The Netherlands, Maastricht, Netherlands; ^7^ Harvard Medical School, Boston, MA, USA; ^8^ Massachusetts General Hospital, Boston, MA, USA; ^9^ Brigham and Women's Hospital, Boston, MA, USA; ^10^ Yale University, New Haven, CT, USA; ^11^ Athinoula A. Martinos Center for Biomedical Imaging, Massachusetts General Hospital, Harvard Medical School, Boston, MA, USA

## Abstract

**Background:**

Brainstem nuclei such as the locus coeruleus (LC) are amongst the earliest regions affected by tau pathology in Alzheimer's disease (AD). The LC's extensive noradrenergic projections shape brain network architecture and its structural integrity is associated with resilience against cognitive decline. However, the role of LC network connectivity in cognitive resilience remains unclear, as does its potential differential impact across distinct population subgroups who might specifically benefit from its augmentation.

**Methods:**

We included 393 cognitively unimpaired Aβ+ individuals (centiloid > 19) from the A4 study who underwent baseline resting‐state fMRI and florbetapir Aβ‐PET as well as longitudinal Preclinical Alzheimer's Cognitive Composite (PACC) assessment. Pearsons's correlation coefficient was used to create maps of LC functional connectivity (LC‐FC) to 454 parcels of the Schaefer‐Tian parcellation which were harmonised across scanners using NeuroCombat. Mixed‐effects models with natural cubic splines (2 DOF) were used to test whether global, Yeo17 network, and individual parcel level LC‐FC moderated PACC decline over time as well as in interaction with centiloid, sex, and APOΕ4 status, controlling for age, education, framewise displacement, treatment, and sex (when not interacted). Analyses were deemed significant following Benjamini‐Hochberg false‐discovery rate correction for multiple comparisons.

**Results:**

The LC showed widespread connectivity that was strongest within the limbic regions, hippocampus, and thalamus (Figure 1a). Greater global LC‐FC was associated with attenuated cognitive decline (Figure 1b, *p* = 0.014), especially at higher levels of Aβ (Figure 1c, *p* <0.001). At the network level, LC‐FC resilience was predominantly related to task‐positive (control and dorsal/ventral attentional) networks, whilst in the default mode network greater LC‐FC was associated with reduced cognitive decline at lower Aβ (Figure 1b/c). The effect of LC‐FC on PACC decline was particularly pronounced in females at high Aβ levels (Figure 2b) and APOE‐e4 carriers (Figure 2c), demonstrating a synergistic effect of sex and genetic risk on LC‐mediated cognitive resilience (Figure 2d).

**Conclusions:**

Our findings further support the role of the LC in cognitive resilience and suggest this particularly manifests from modulation of task‐positive attentional and frontoparietal control networks. Moreover, female e4‐carriers exhibit more pronounced attenuation of cognitive decline, suggesting they may especially benefit from augmentation of noradrenergic function.